# Supplementary material for: Extreme amyloid polymorphism in Staphylococcus aureus virulent PSMα peptides
Source: Nat Commun. 2018 Aug 29;9:3512. doi: 10.1038/s41467-018-05490-0 (PMC6115460; doi:10.1038/s41467-018-05490-0)
Supplement: Supplementary file 1 — Supplementary Information [file 41467_2018_5490_MOESM1_ESM.pdf]

Extreme Amyloid Polymorphism in *Staphylococcus aureus* Virulent PSMα Peptides

Salinas et al.

Supplementary Figure 1: Amyloidogenic segments in *S. aureus* PSMαs

a

PSMα1 (H9BRQ5) MGIIAG**IIKVIK**SLIEQFTGK

PSMα3 (H9BRQ7) MEFVAK**LFKFFK**DLLGKFLGNN

PSMα4 (H9BRQ8) MAIVGT**IIKIIK**AIIDIFAK

b

Peptide pairs	Identity
PSMα1 - PSMα3	32%
PSMα1 - PSMα4	48%
PSMα3 - PSMα4	23%

(a) The sequences of *S. aureus* PSMα1, PSMα3 and PSMα4 (UniProt accession number is indicated in parentheses). Segments marked in bold were predicted to have amyloidogenic propensity based on computational methods[1-5], and were selected for structural characterization. **(b)** Percentage of sequence identity between pairs of PSMα.

Supplementary Figure 2. Thioflavin T binding and fibrillation kinetics of PSMα1 and PSMα4

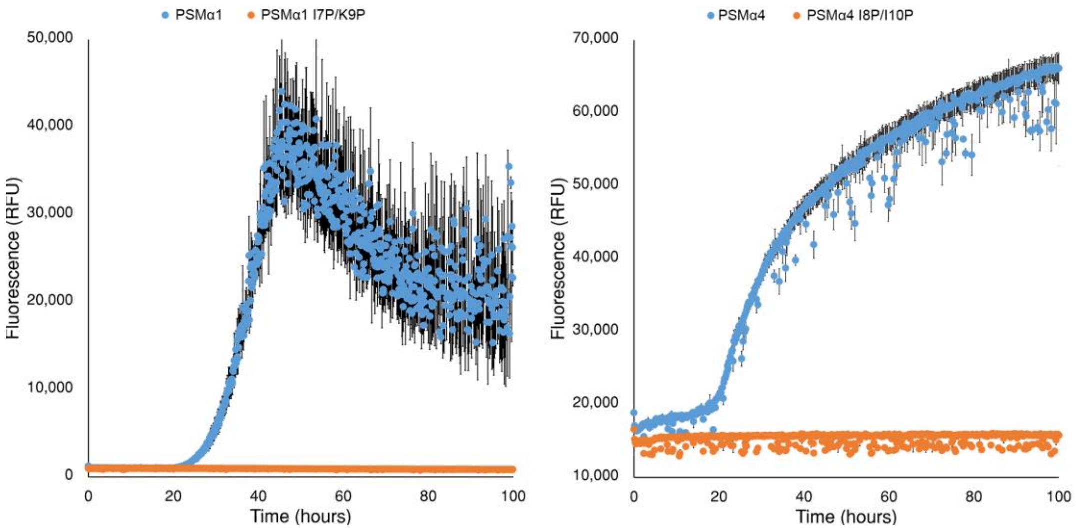

Fibrillation kinetics of PSMα1, PSMα4 and mutants that sustained substitution of two residues within the predicted amyloidogenic region to proline residues (PSMα1 I7P/K9P and PSMα4 I8P/I10P). The fibrillation reaction contained 50 µM peptide and 200 µM Thioflavin T. PSMα1 and PSMα4 showed rapid fibrillation following a ~20 h lag time. The mutants showed no ThT binding. The graph shows mean fluorescence readings of triplicate ThT measurements. Calculated standard errors of the mean are presented as error bars.

Supplementary Figure 3. Steric-zipper structure of PSMα4 IIKIIK

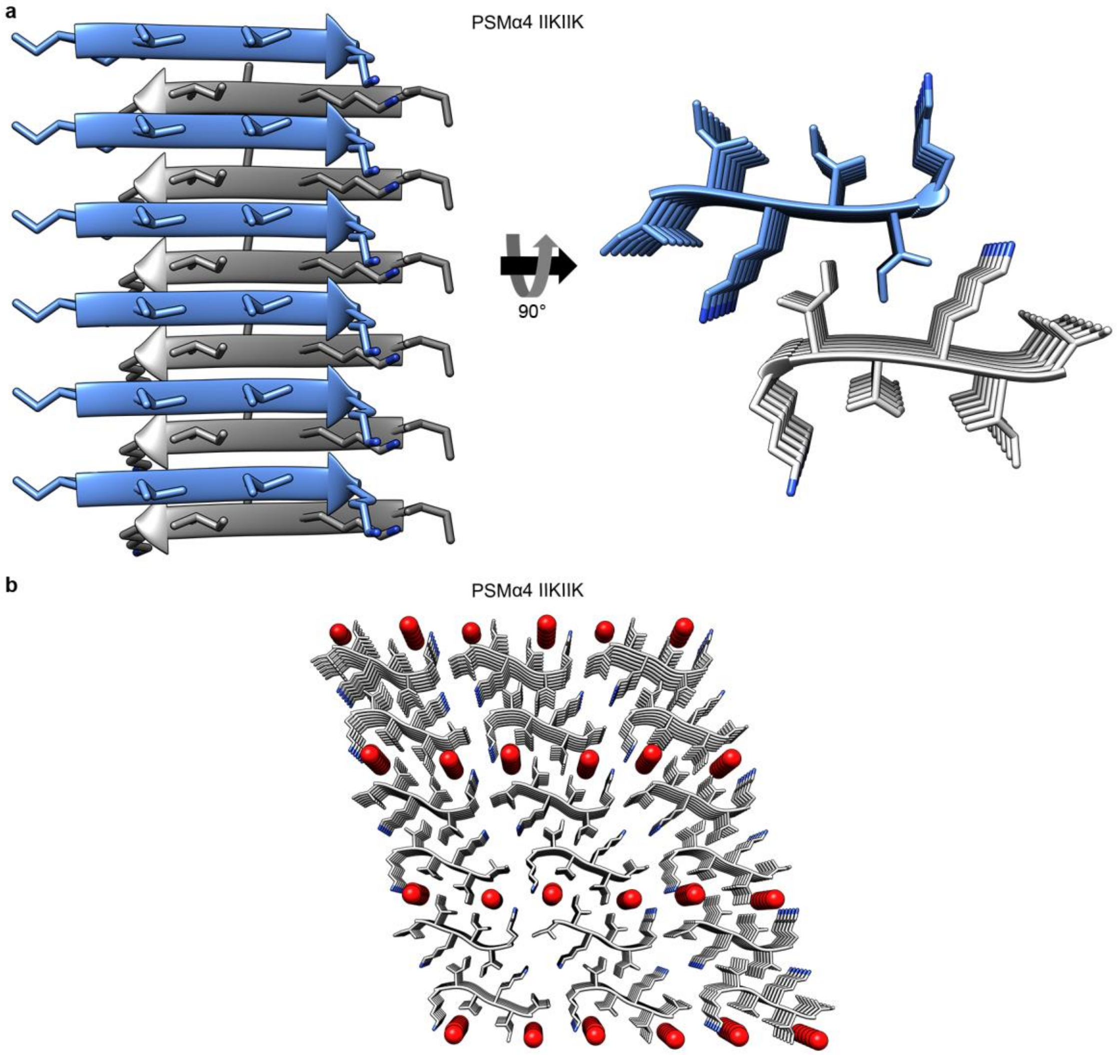

(a) The structure of PSMα4-IIKIIK determined at 1.6Å resolution included pairs of β-sheets which are the basic unit of the fibril; side-chains protruding from the two β-sheets intermesh to form a dry, tightly self-complementing steric zipper bonding the sheets. The β-sheets are composed of parallel β-strands. In the left panel, the view is perpendicular to the fibril axis and the β-strands run horizontally. In the right panel, the view is down the fibril axis. The segments are shown in ribbon representation, with side chains shown as sticks. The carbons within each β-sheet are colored either gray or light blue, and nitrogen atoms in side chains are colored blue. The structure of IIKIIK is highly similar to the structure of IIKVIK from PSMα1 (Fig. 2). **(b)** The crystal packing of IIKIIK from PSMα4 is depicted down the fibril axis showing alternating dry and wet interfaces. Residues colored by atom-type are represented as sticks, and water molecules are in red spheres.

Supplementary Figure 4. Antibacterial activity of LFKFFK and KLFKFFK in solution

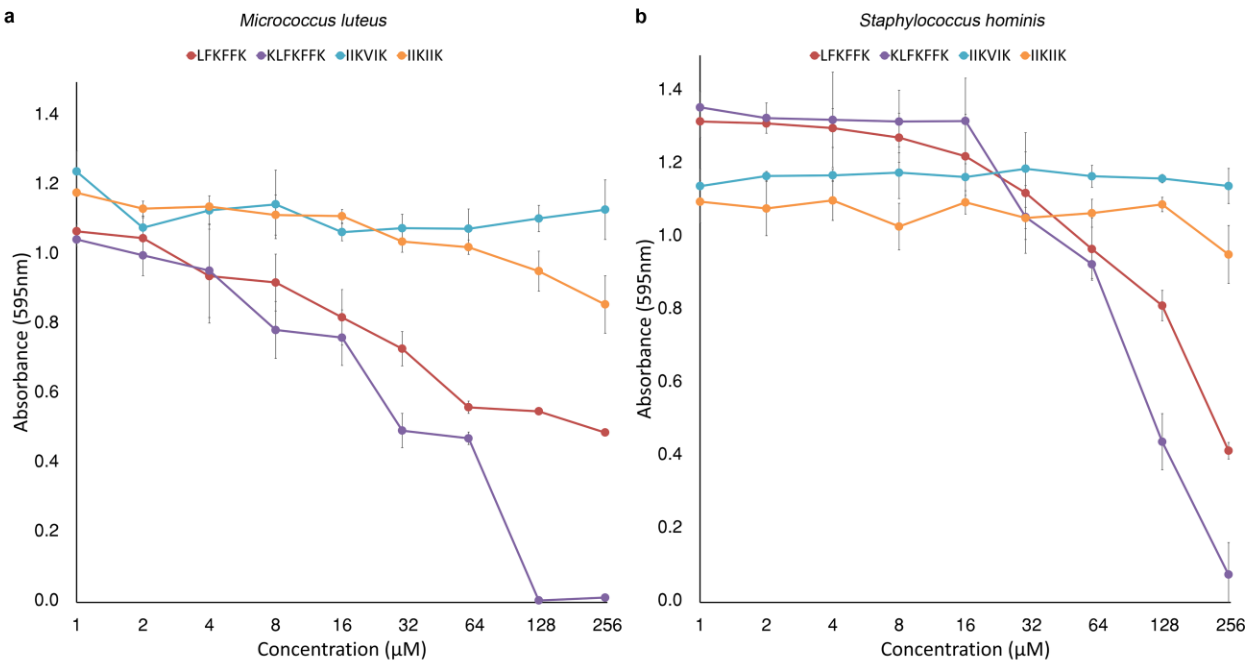

The LFKFFK and KLFKFFK peptides from PSMα3 inhibited the growth of *M. luteus* **(a)** and *S. hominis* **(b)** in a dose-dependent manner. The graphs show the maximal optical density (OD) of *M. luteus* culture at t=18 hours **(a)**, or *S. hominis* culture at t=8 hours **(b)** in the presence of different peptide concentrations. The steric-zipper-forming segments, IIKVIK from PSMα1 and IIKIIK from PSMα4, did not show a significant antibacterial effect on either species. The mean OD values and error bars represent triplicate measurements that were averaged from three individual experiments performed on different days. Calculated standard errors of the mean are presented as error bars.

Supplementary Figure 5. Attenuated total internal reflection Fourier transform infrared spectra of PSMαs segments

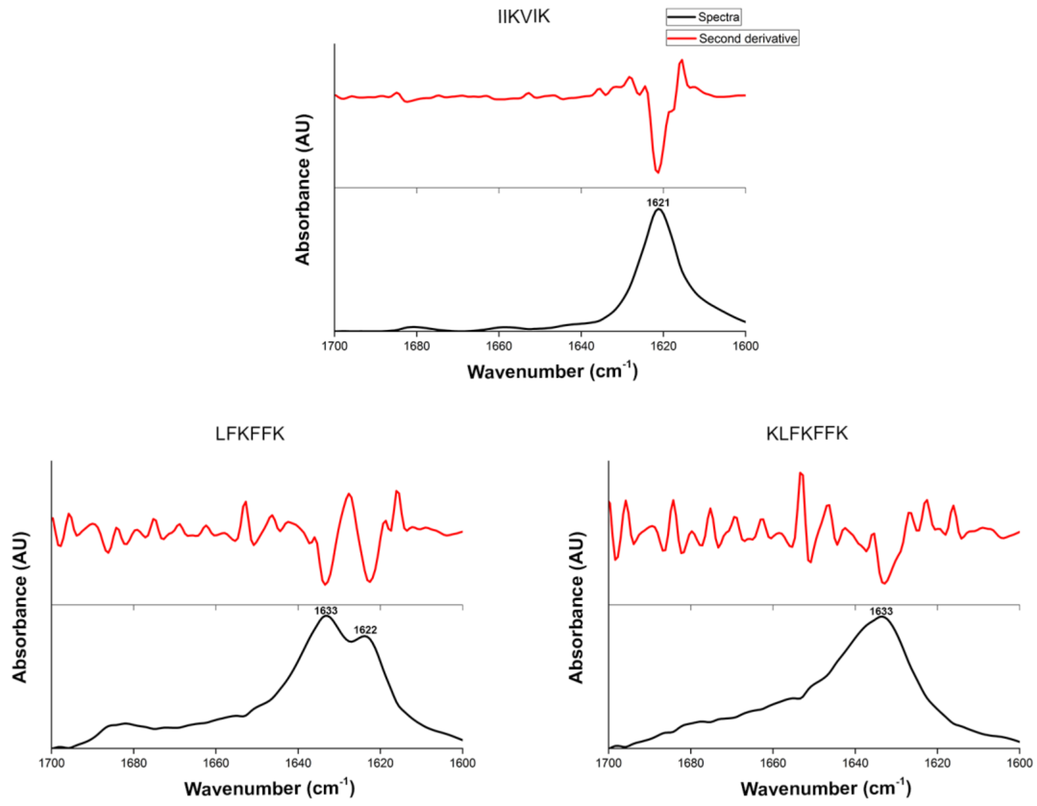

Attenuated total internal reflection Fourier transform infrared (ATR-FTIR) spectroscopy of the amide I' region (1600-1700 cm^{-1}) of fibrils of PSMα segments. The canonical steric-zipper forming spine segment PSMα1-IIKVIK shows a peak at 1621 cm^{-1} corresponding to rigid amyloid fibrils[6-8]. In contrast, PSMα3-KLFKFFK shows a peak at 1633 cm^{-1} and PSMα3-LFKFFK shows two main peaks at 1622 cm^{-1} and 1633 cm^{-1}, with the latter indicating on more disordered fibrils with absorbance which is typical of the bent β-sheets in proteins[6-8].

Supplementary Figure 6. Extreme polymorphism of the self-assembling PSMαs

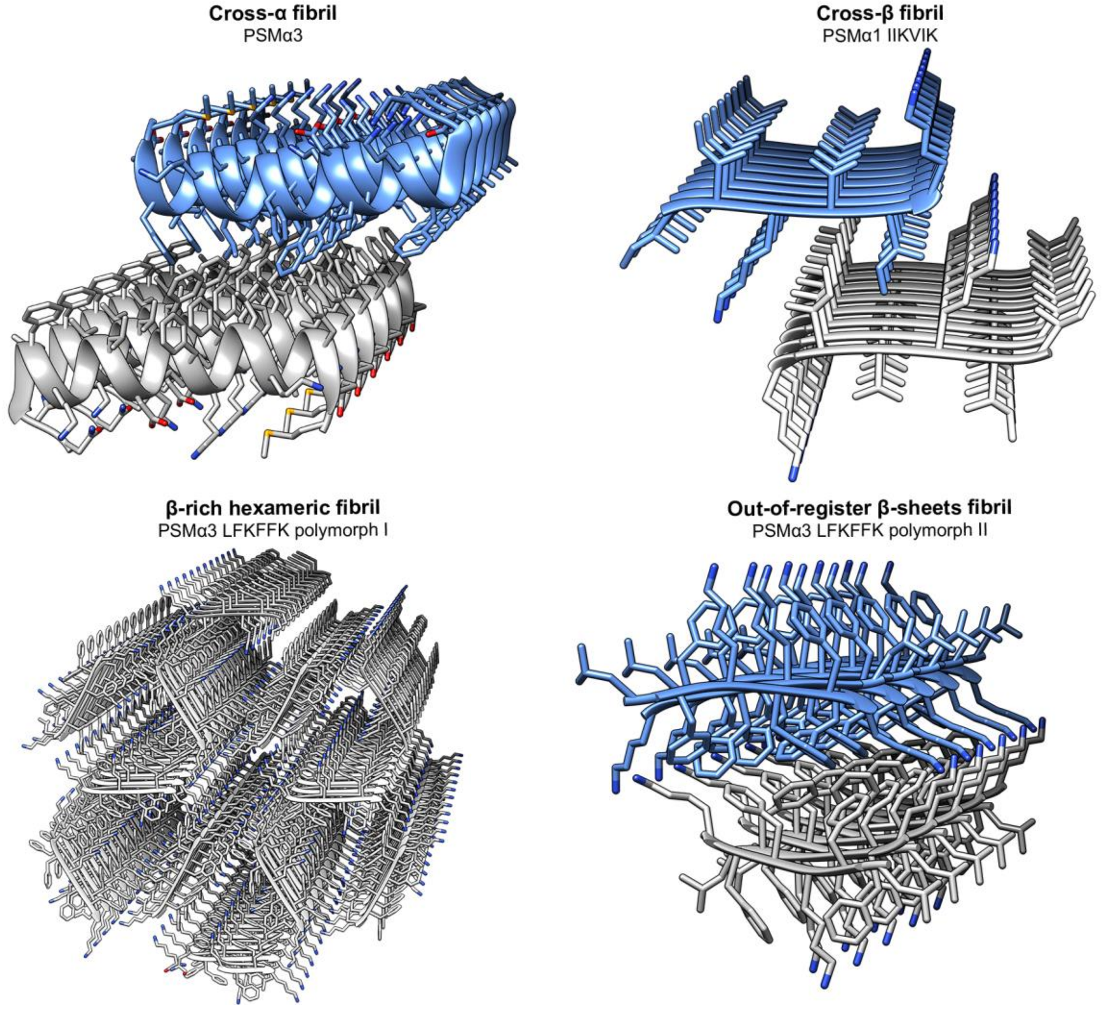

The cross-α amyloid-like fibrils of PSMα3[9] (top left panel) as well as the cross-β steric-zipper fibrils of PSMα1-IIKVIK (top right panel) form pairs of tightly mated parallel sheets running along the fibril axis. Here only six layers are shown. Individual molecules are oriented perpendicular to the fibril axis. The two polymorphs of the PSMα3 LFKFFK segment are shown in the bottom panel. One polymorph forms hexameric configuration of antiparallel β-sheets, yielding nanotubes along the fibril (bottom left). The second polymorph form out-of-register mating pairs of antiparallel β-sheets (bottom right). The peptides are shown in ribbon representation with side chains shown as sticks. The carbons within each sheet are colored either gray or light blue, with heteroatoms in side chains colored by atom type (nitrogen in blue, oxygen in red, and sulfur in yellow).

Supplementary Table 1. Quantitative measures of amyloid stability based on features of the crystal structures of PSMα

	**PSMα1<br>IIKVIK**	**PSMα4<br>IIKIIK**	**PSMα3<br>LFKFFK<br>Polymorph I**	**PSMα3<br>LFKFFK<br>Polymorph II**
**Shape complementarity**	0.89	0.89	0.79	0.82
**Inter-strand distance along the sheet**	4.80 Å	4.83 Å	4.82 Å<br>(between antiparallel β-strands)	4.80 Å<br>(between antiparallel β-strands)
**Area buried**	One molecule against an opposite sheet:<br>532 $Å^2$<br>(28%)[a]<br>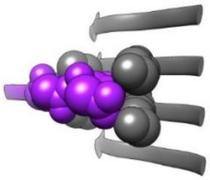	One molecule against an opposite sheet:<br>510 $Å^2$<br>(27%)[a]<br>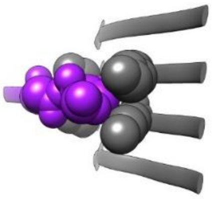	One molecule against two opposite sheets in the trimer:<br>Strand 1[b]:<br>223 $Å^2$<br>(11%)[a]<br>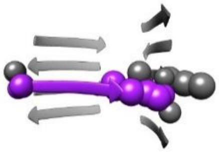<br>Strand 2[b]:<br>281 $Å^2$<br>(13%)[a]<br>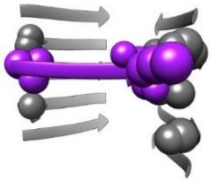	One molecule against an opposite sheet:<br>Strand 1[b]:<br>414 $Å^2$<br>(20%)[a]<br>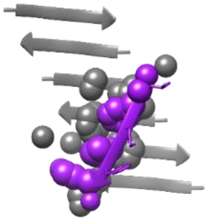<br>Strand 2[b]:<br>438 $Å^2$<br>(21%)[a]<br>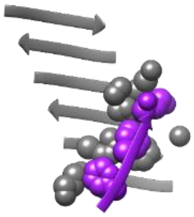

	One molecule within pairs of sheets:<br><br>1343 Å^2 (72%)[a]<br>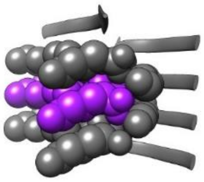	One molecule within pairs of sheets:<br><br>1338 Å^2 (71%)[a]<br>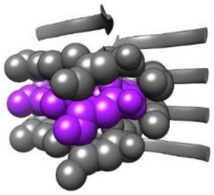	One molecule within a trimer:<br><br>Strand 1[b]: 1342 Å^2 (62%)[a]<br>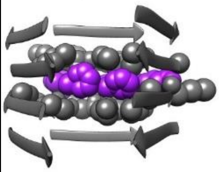<br><br>Strand 2[b]: 1380 Å^2 (64%)[a]<br>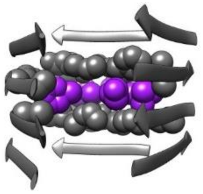	One molecule within pairs of sheets:<br><br>Strand 1[b]: 1305 Å^2 (62%)[a]<br>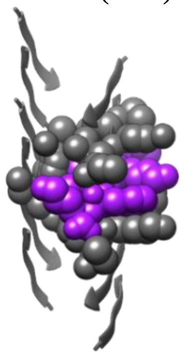<br><br>Strand 2[b]: 1329 Å^2 (63%)[a]<br>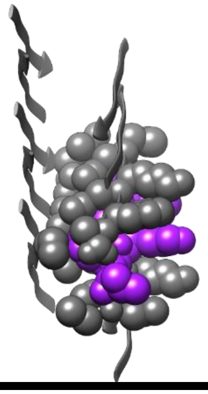

	**NNQQNY**	**VQIVYK**	**KLVFFA**
**Shape complementarity**	0.86	0.72	0.59
**Inter-strand distance along the sheet**	4.87 Å	4.86 Å	4.79 Å (between antiparallel β-strands)

**Area buried**	One molecule against an opposite sheet:<br><br>469 Å^2 (27%)[a]<br>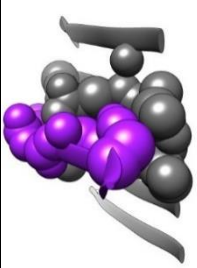	One molecule against an opposite sheet:<br><br>325 Å^2 (17%)[a]<br>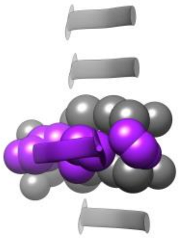	One molecule against an opposite sheet:<br><br>Strand 1[b]:<br>242 Å^2<br>(13%)[a]<br>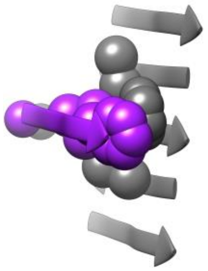<br>Strand 2[b]:<br>334 Å^2<br>(18 %)[a]<br>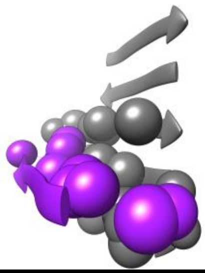
	One molecule within pairs of sheets:<br><br>1268 Å^2 (75%)[a]<br>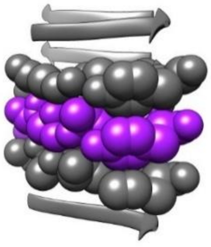	One molecule within pairs of sheets:<br><br>1352 Å^2 (69%)[a]<br>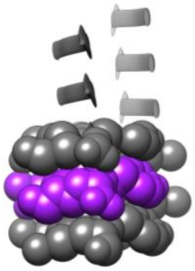	One molecule within pairs of sheets:<br><br>Strand 1[b]:<br>1252 Å^2 (65%)[a]<br>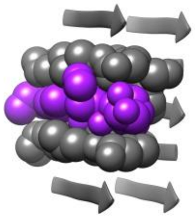<br>Strand 2[b]:<br>1344 Å^2 (70%)[a]<br>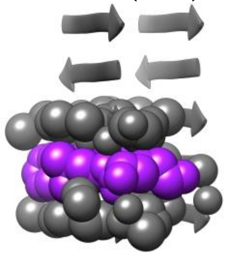

Since steric-zipper fibrils are unusual in that pairs of β-sheets mate more closely than the adjoining surfaces in other protein complexes, quantitative measures of amyloid stability are based on solvent-accessible surface area buried at the interface between the mating sheets, and shape complementarity indicating on the closeness of fit of two protein surfaces[10]. Shape complementarity of zero indicates no complementarity of the two surfaces and approaches 1.0 for atomic surfaces that fit perfectly together[11].

The values of shape complementarity and surface area buried calculated for the PSMα structures are compared with those of the VQIVYK segment from the tau protein involved in Alzheimer's disease (PDB code 2ON9)[12], KLVFFA segment from amyloid-β involved in Alzheimer's disease (PDB code 2Y2A)[13], and the NNQQNY segment from yeast prion Sup35 (PDB code 1YJO)[14]. NNQQNY shows one of the highest values of shape complementarity and surface area buried among steric zipper structures[12].

[a] The number depicted is the percentage of the total accessible surface area of one strand (colored purple) that is in contact with the surrounding depicted strands (colored grey).

[b] LFKFFK and KLVFFA assemble into antiparallel β-sheets, hence each of the antiparallel β-strands forms a difference interface that was calculated separately.

Supplementary References

1 Goldschmidt, L., Teng, P. K., Riek, R. & Eisenberg, D. Identifying the amylome, proteins capable of forming amyloid-like fibrils. *Proc. Natl. Acad. Sci. U.S.A.* **107**, 3487-3492, (2010).

2 Tartaglia, G. G. *et al.* Prediction of aggregation-prone regions in structured proteins. *J. Mol. Biol.* **380**, 425-436, (2008).

3 Fernandez-Escamilla, A. M., Rousseau, F., Schymkowitz, J. & Serrano, L. Prediction of sequence-dependent and mutational effects on the aggregation of peptides and proteins. *Nat. Biotechnol.* **22**, 1302-1306, (2004).

4 Maurer-Stroh, S. *et al.* Exploring the sequence determinants of amyloid structure using position-specific scoring matrices. *Nat. Methods* **7**, 237-242, (2010).

5 Rousseau, F., Schymkowitz, J. & Serrano, L. Protein aggregation and amyloidosis: confusion of the kinds? *Curr. Opin. Struc. Biol.* **16**, 118-126, (2006).

6 Sarroukh, R., Goormaghtigh, E., Ruysschaert, J. M. & Raussens, V. ATR-FTIR: a "rejuvenated" tool to investigate amyloid proteins. *Biochim. Biophys. Acta* **1828**, 2328-2338, (2013).

7 Zandomeneghi, G., Krebs, M. R., McCammon, M. G. & Fandrich, M. FTIR reveals structural differences between native beta-sheet proteins and amyloid fibrils. *Protein Sci.* **13**, 3314-3321, (2004).

8 Moran, S. D. & Zanni, M. T. How to Get Insight into Amyloid Structure and Formation from Infrared Spectroscopy. *J. Phys. Chem. Lett.* **5**, 1984-1993, (2014).

9 Tayeb-Fligelman, E. *et al.* The cytotoxic Staphylococcus aureus PSMalpha3 reveals a cross-alpha amyloid-like fibril. *Science* **355**, 831-833, (2017).

10 Eisenberg, D. S. & Sawaya, M. R. Structural Studies of Amyloid Proteins at the Molecular Level. *Annu. Rev. Biochem.* **86**, 69-95, (2017).

11 Lawrence, M. C. & Colman, P. M. Shape complementarity at protein/protein interfaces. *J. Mol. Biol.* **234**, 946-950, (1993).

12 Sawaya, M. R. *et al.* Atomic structures of amyloid cross-beta spines reveal varied steric zippers. *Nature* **447**, 453-457, (2007).

13 Colletier, J.-P. *et al.* Molecular basis for amyloid-beta polymorphism. *Proc. Natl. Acad. Sci. U.S.A.* **108**, 16938-16943, (2011).

14 Nelson, R. *et al.* Structure of the cross-beta spine of amyloid-like fibrils. *Nature* **435**, 773-778, (2005).
